# Long-Term Prospective Follow-Up of Spinal Interventional Pain Procedures and Physical Therapy Compliance on Outcomes in Chronic Pain Patients

**DOI:** 10.3390/jcm14176271

**Published:** 2025-09-05

**Authors:** Wael Saleem, Jo Ann LeQuang, Muntaha Elayyan, Mustafa Rezk, Zeineb Fourati, Ahmad Hajaj, Mohammed Orompurath, Shaif Jarallah, Flaminia Coluzzi, Joseph V. Pergolizzi

**Affiliations:** 1 Department of Anesthesiology/ICU and Perioperative Medicine, Hamad Medical Corporation, Doha P.O. BOX 3050, Qatar; mrezk@hamad.qa (M.R.); zfourati@hamad.qa (Z.F.); morompurath@hamad.qa (M.O.); 2 Department of Scientific Communication, NEMA Research, Naples, FL 34108, USA; joannlequang@gmail.com (J.A.L.); jpergolizzi@nemaresearch.net (J.V.P.J.); 3 Department of Pain Management, NEMA Research, Naples, FL 34108, USA; 4 Department of Nursing and Midwifery, Hamad Medical Corporation, Doha P.O. Box 2713, Qatar; melayyan@hamad.qa (M.E.); ahajaj1@hamad.qa (A.H.); 5 Department of Finance and Economics, Collage of Business Economics, Qatar University, Doha P.O. Box 2713, Qatar; shaif@qu.edu.qa; 6 Department of Surgical and Medical Sciences and Translational Medicine, Sapienza University of Rome, 00189 Rome, Italy; flaminia.coluzzi@uniroma1.it; 7 Unit Anesthesia, Intensive Care and Pain Therapy, Sant’Andrea University Hospital, 00189 Rome, Italy

**Keywords:** chronic back pain, interventional pain procedures, EQ5D, NRS, ODI, physical therapy compliance

## Abstract

**Background/Objective:** The long-term implications of spinal interventional pain procedures (IPPs) remain underexplored. This study aimed to evaluate pain intensity, overall health quality, and disability over a four-year period following an IPP, while also assessing physical therapy (PT) compliance, the need for repeated procedures, and analgesic consumption. **Methods:** A prospective observational study with retrospective registration was conducted on patients with chronic spinal pain at Hamad Medical Corporation (HMC), Qatar. Patients were assessed using the Numeric Rating Scale (NRS), the Oswestry Disability Index (ODI), and the EuroQol 5-Dimension Index (EQ-5D). Additional tools included the EQ-Health Visual Analog Scale and the Patient Global Impression of Change (PGIC). Follow-ups were performed at 6, 12, 24, 36, and 48 months. **Results:** A total of 766 patients were initially recruited, of whom 174 met the inclusion criteria. All patients demonstrated significant improvement across all outcome measures compared to the baseline. No significant differences were observed in pain or functional outcomes between PT-compliant and non-compliant groups. By the end of this study, 56.9% of patients reported reduced analgesic use, while 43.1% reported increased use. The patient satisfaction data showed that 55% (62/113) of those receiving a single IPP were satisfied, compared to 54% (33/61) in the multiple IPP group. The PGIC satisfaction scores revealed no statistically significant differences (*p* = 1). Overall health status, as measured by the EQ-5D Health scale, also showed no meaningful differences between groups. **Conclusions:** Despite several limitations, patients reported sustained improvement and satisfaction four years post-IPP. PT compliance did not appear to significantly influence long-term outcomes. Further research is needed to identify factors affecting post-IPP recovery and medication usage.

## 1. Introduction

Of all chronic pain conditions globally, none is more prevalent or more disruptive to daily activities and workplace attendance than chronic back pain (CBP) [1,2]. Central sensitization mechanisms can lead to the transition from acute to chronic back pain, making it significantly more difficult to treat [3]. The global burden of back pain constitutes a public health crisis; the World Health Organization estimates that 619 million people suffer from back pain worldwide, a proportion of whom go on to develop CBP [4]. The societal and healthcare impact of CBP, including resource allocation, lost productivity, diminished quality of life, and direct economic costs, is substantial and often difficult to quantify.

While no single “gold standard” treatment exists for CBP, conservative approaches currently represent the foundation of management. This preference is partly driven by the potential risks and adverse effects associated with more invasive methods [5,6]. Patients who are managed conservatively often pursue self-directed strategies to alleviate symptoms, including over-the-counter medications, posture-corrective footwear or bedding, physical exercise, massage, and various complementary and alternative therapies [7].

Interventional pain procedures (IPPs) have become pivotal in managing chronic back pain, particularly when conservative treatments fail to provide adequate relief. These procedures aim to disrupt aberrant pain signaling pathways, thereby alleviating pain and improving function. Techniques such as transforaminal epidural steroid injections, medial branch blocks, and radiofrequency ablations target specific neural structures involved in pain transmission [8,9,10]. For example, radiofrequency ablation of the medial branch nerves can interrupt nociceptive input from facet joints, resulting in significant pain reductions. Similarly, epidural steroid injections can reduce inflammation around nerve roots, alleviating radicular pain. The efficacy of these interventions has been supported by multiple studies demonstrating consistent improvements in pain scores and functional outcomes [11,12]. The analgesic relief offered by these procedures may last from several months to multiple years, depending on factors such as patient adherence to therapy, diagnostic accuracy, type of pain, psychosocial influences, age, and overall frailty [8,9,10]. As a result, clinicians may find it challenging to predict treatment duration or the extent of analgesic benefit for individual patients.

Physical therapy (PT) remains a cornerstone in the management of chronic low back pain (cLBP), particularly during rehabilitation following interventional procedures. PT improves core strength, flexibility, and posture, supporting functional recovery and potentially reducing the risk of recurrence or chronicity. A recent level I Bayesian network meta-analysis by Baroncini et al. [13] assessed various physiotherapeutic and non-conventional approaches for cLBP, finding that while multiple interventions demonstrated benefits, their overall effectiveness varied, reinforcing the importance of individualized treatment planning. In contrast, a systematic review and meta-analysis by Migliorini et al. [14] reported that the addition of educational interventions to physiotherapy did not significantly improve pain or disability outcomes in patients with chronic non-specific low back pain. Despite these insights, PT compliance in real-world settings remains inconsistent and is often influenced by factors such as patient motivation, access to care, and perceived treatment value. This study, therefore, aims to evaluate the long-term role of PT compliance alongside interventional pain procedures in the management of chronic back pain.

The objective of this study was to longitudinally assess pain levels, overall well-being, and functional outcomes in patients with chronic back pain (CBP) undergoing one of three specific interventional pain procedures (IPPs). These procedures included transforaminal epidural steroid injections for nerve blockades, medial branch (facet joint) interventions for diagnostic evaluations and/or radiofrequency ablation, and sacroiliac joint procedures for both diagnosis and ablation. Given the potential influence of physiotherapy (PT) adherence on outcomes, This study also evaluated PT-compliant patients to determine their need for additional IPPs. The effectiveness of pain relief was assessed in part through changes in analgesic consumption.

## 2. Materials and Methods

This was a prospective observational study with retrospective registration, conducted following ethical approval from the Hamad Medical Corporation’s Ethical Committee (MRC-01-19-312). All procedures took place between January and December 2017 and involved 766 patients diagnosed with chronic back pain. Participants were followed for a duration of 48 months. The extended timeline for publication was due to the complexity of long-term data collection, ethical approval processes, the COVID-19 pandemic, and challenges related to patient communication and coordination.

Participants were eligible for inclusion if they were between 18 and 80 years of age and had experienced persistent chronic back pain for at least six consecutive months, with minimal or no improvement following conventional treatments, including physical therapy. Only patients indicated for spinal interventional pain procedures (IPPs) targeting the facet joints, sacroiliac joints, or nerve root pathology were included. Although chronic pain is often defined as lasting more than three months, a six-month threshold was used in this study to ensure the inclusion of patients with well-established, treatment-resistant conditions, thereby improving cohort specificity.

Exclusion criteria included the presence of spinal red flags (e.g., infection, tumor, fracture, or neurological disease), peripheral neuropathic conditions, peripheral vascular disease, or mental illness. Patients who failed to complete all scheduled follow-up assessments over the 48-month study period were also excluded.

The exclusion criteria were deliberately comprehensive to minimize confounding factors that could obscure spinal pathology or impact treatment adherence. Conditions such as musculoskeletal disorders, peripheral vascular disease, or neuropathic comorbidities were excluded, as they may mimic or mask spinal radiculopathy, complicating clinical interpretation of pain origin or compliance with physical therapy. Likewise, patients with mental/cognitive concerns (e.g., depression, dementia) were excluded due to the potential for reduced compliance with physiotherapy and long-term follow-up, as well as difficulty accurately reporting symptoms or outcomes. This stringent selection process ensured that this study focused on patients with well-defined, spine-related chronic pain amenable to both interventional procedures and physical therapy.

Prior to undergoing the procedures, all patients received a detailed explanation of the interventions and their potential side effects. Informed consent was obtained from each participant. The specific procedure performed was determined based on individual clinical indication: some patients received only transforaminal epidural steroid injections, while others underwent an initial diagnostic steroid block followed by radiofrequency (RF) treatment. RF ablation was carried out using standard, evidence-based techniques [15]. Each intervention was tailored to the patient’s clinical presentation and anatomical target, including medial and lateral branch blocks, sacroiliac joint blocks, and transforaminal epidural steroid injections (Table 1).

To assess the efficacy of interventional pain procedures (IPPs), this study employed the Numeric Rating Scale (NRS), the EuroQol 5-Dimension 5-Level (EQ-5D-5L), and the Oswestry Disability Index (ODI). These questionnaires were administered at the baseline and at each follow-up interval.

Follow-up assessments were conducted starting from the date of the first IPP, either via telephone consultations or outpatient clinic visits, at 6, 12, 24, 36, and 48 months. Any additional procedures performed during the study period were documented but did not reset the follow-up timeline. In some cases, follow-up data were also obtained from patients’ electronic medical records. All follow-up was conducted by a team blinded to the specific procedures performed. Patients were referred to initiate or continue physiotherapy as part of their management plan. Repeat IPPs were administered in the same anatomical region if clinically indicated and were documented for further analysis. Final outcomes included patient-reported satisfaction and EQ health index scores.

PT compliance was defined as full participation in all scheduled sessions, verified through both physiotherapy records and patient interviews. Patients who discontinued PT after initial participation were classified as non-compliant, since compliance was defined across the entire 4-year follow-up period. Physiotherapy sessions included posture and positioning exercises, muscle stretching, and application of thermal or electrical modalities. A standard PT course was defined as three sessions per week over a six-week period. Based on physician recommendations, patients typically received two to three courses of PT annually.

Medication use was tracked throughout this study. Patients were asked to report their use of prescribed pain medications, which was cross-verified with their electronic prescription refill records. Monitored medications included nonsteroidal anti-inflammatory drugs (NSAIDs), acetaminophen (paracetamol), anticonvulsants for neuropathic pain, antidepressants, muscle relaxants, NMDA receptor antagonists, and opioids.

### Statistics

Data were collected as both continuous and nominal variables. Normality was assessed using the Shapiro–Wilk and Kolmogorov–Smirnov tests to ensure robustness across varying sample distributions. For continuous variables, descriptive statistics were reported as medians with interquartile ranges (IQRs). For nominal variables, frequencies and valid percentages were presented.

To compare differences between two independent groups, the Mann–Whitney U test was used. For comparisons among more than two independent groups, the Kruskal–Wallis test was employed. For related samples, the Wilcoxon Signed-Rank Test was used when comparing two time points, and Dunn’s test was used for multiple related groups. A *p*-value of <0.05 was considered statistically significant. Where appropriate, means and standard deviations were also reported. All analyses were performed using IBM SPSS Statistics, Version 22.

Normality Testing:

Normality was assessed using both the Shapiro–Wilk and Kolmogorov–Smirnov tests. A *p*-value of <0.05 in both tests indicated that the dataset did not follow a normal distribution.

Comparative Analyses:

The Mann–Whitney U test was applied to investigate differences in NRS, EQ-5D, and ODI scores based on the number of IPPs (single vs. multiple), medication use patterns, and sex. The Kruskal–Wallis H test followed by Dunn’s post hoc test was used to compare physical therapy (PT) compliance across multiple IPP subgroups. While the Kruskal–Wallis test indicated a significant overall difference (*p* = 0.045), Dunn’s post hoc comparisons showed no statistically significant pairwise differences after Bonferroni correction, although the comparison between PT-compliant patients and those receiving multiple IPPs had a borderline unadjusted *p*-value (*p* = 0.032). In contrast, a separate Kruskal–Wallis test yielded no significant group differences (*p* = 0.888), and Dunn’s test confirmed no significant pairwise results.

To evaluate the relationship between PT compliance and medication use, Kruskal–Wallis tests followed by Dunn’s post hoc analysis were conducted to compare NRS, EQ-5D, and ODI scores across PT and medication status groups at multiple time points (6, 12, 24, 36, and 48 months). No statistically significant differences were found at any time point.

Finally, the Wilcoxon Signed-Rank Test was used to examine changes in NRS, EQ-5D, and ODI scores over time within PT-compliant and non-compliant groups

## 3. Results

**Figure 1:**
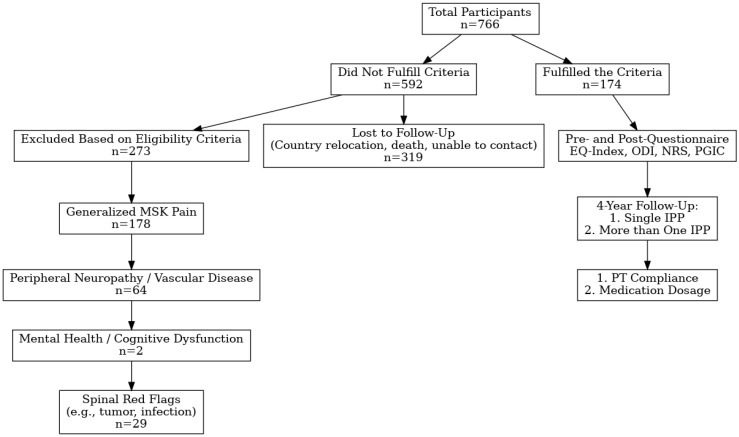
Flowchart of participant inclusion and follow-up in this study. Abbrev: EQIndex, Euro Qual Index; IPP, interventional pain procedure; MSK, musculoskeletal; and PT, physical therapy.

The study cohort comprised 766 patients who underwent a spine-related interventional pain procedure (IPP) during the calendar year 2017. Patients who did not meet all inclusion criteria or failed to complete the full follow-up schedule were excluded, resulting in a final analytic sample of 174 participants (Figure 1). The demographic and clinical characteristics of the study cohort are presented in Table 1. Facet-related arthropathy was the most common diagnosis, accounting for 38.5% of cases. Most participants (60.9%) received only one IPP.

At the 48-month follow-up, patient medication use was reassessed: 56.9% of patients had decreased their analgesic intake, while 43.1% had increased consumption. Adherence to physical therapy was also evaluated at the final follow-up, revealing that 60.3% of patients were non-compliant with their prescribed PT regimen.

### 3.1. The IPP Effect on NRS, EQ5D, and ODI

**Figure 2:**
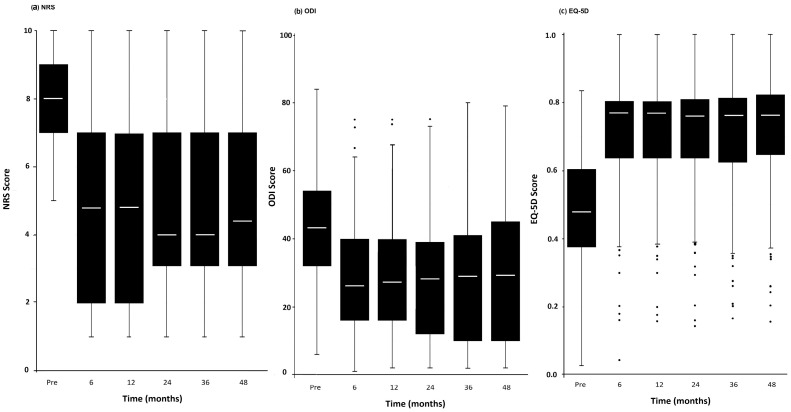
The trend responses of IPPs in EQ5D (**a**), NRS (**b**), and ODI (**c**). This trend is independent of the total number of procedures. Dots below the bars indicate statistical outliers.

The Mann–Whitney U nonparametric test was used to calculate specific Z scores and assess differences between pre- and post-intervention time points. A broad range of individual responses was observed across all follow-up intervals. Nonetheless, statistically significant improvements (*p* < 0.05) were noted in all three outcome measures—NRS, EQ-5D, and ODI—when comparing the baseline scores to post-IPP scores across each time period. These results are illustrated in Figure 2 and detailed in Table 2.

### 3.2. Physical Therapy Compliance

The effect of physical therapy (PT) compliance on patient outcomes was evaluated by comparing PT-compliant and non-compliant groups (Table 3). Statistically significant improvements in NRS scores were observed between the baseline and 6 months, as well as between 12 and 24 months, in both groups—with more favorable trends noted in the PT-compliant group. No significant changes were found between 6 and 12 months, 24 and 36 months, or 36 and 48 months.

In terms of quality of life (measured by EQ-5D), significant differences were detected between the baseline and 6 months, as well as between 24 and 36 months and between 36 and 48 months in both groups. However, no significant changes were found between 6 and 12 months or between 12 and 24 months.

**Figure 3:**
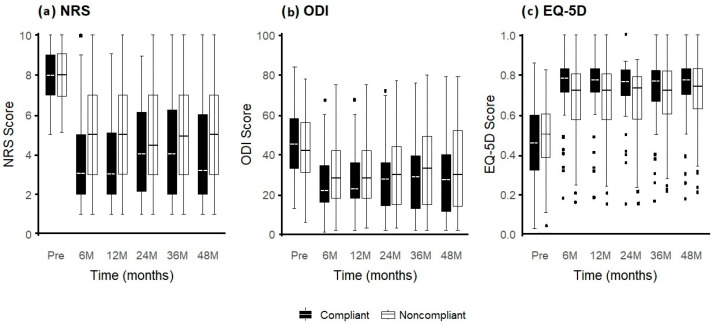
The trend responses of IPPs in relation to PT compliance: EQ5D (**a**) NRS (**b**), and ODI (**c**). This trend is independent of the total number of procedures. Dots below the bars indicate statistical outliers.

Using the Oswestry Disability Index (ODI), significant improvements were observed between the baseline and 6 months, as well as between 6 and 12 months, and 24 and 36 months for both PT-compliant and non-compliant groups. A marginal, nonsignificant change was noted between 12 and 24 months. No significant differences were observed between 36 and 48 months (Figure 3).

### 3.3. Number of IPPs

An analysis of EQ-5D and ODI scores between patient cohorts revealed no statistically significant differences (*p* > 0.05), as shown in Table 4. When evaluating the number of procedures in relation to the three main outcome measures (NRS, EQ-5D, and ODI), no significant differences were found in the NRS scores between patients who underwent a single IPP and those who received multiple IPPs over the 4-year follow-up period. Similarly, the EQ-5D and ODI scores demonstrated no statistically differences among the groups (*p* > 0.05).

### 3.4. Medication Consumption

No significant differences were observed between patients who decreased their medication use and those who increased it, across any of the outcome measures or time points, as all *p*-values exceeded the significance threshold of 0.05.

### 3.5. Effect of PT on the Number of IPPs

A comparison between PT-compliant patients and those who received more than one IPP revealed a marginally significant difference; however, this did not remain statistically significant after correction for multiple comparisons. No significant differences were found between the PT-non-compliant patients and either IPP group (Table 5). These findings suggest that further studies with larger sample sizes may be required to clarify potential associations.

A similar trend was noted in the comparison between PT-non-compliant patients and medication status groups. While a marginally significant difference was initially observed, it did not remain significant after adjustment for multiple comparisons. These results likewise warrant validation in larger cohorts.

### 3.6. Effect of PT Compliance on Medication Use

No significant differences were observed between PT-compliant and non-compliant patients with respect to changes in medication consumption. For the group that reduced their medication use, the Z-value was 1.038 (P_unadj = 0.299; P_adj = 0.898). For the group that increased their medication use, the Z-value was 0.263 (P_unadj = 0.792; P_adj = 1). These findings suggest that PT compliance did not significantly impact medication usage patterns after adjustment for multiple comparisons.

### 3.7. Effect of Sex on Number of Procedures

When examining the relationship between sex and the number of procedures performed, no significant association was found. A *p*-value of 0.815 indicates that sex alone did not appear to influence whether patients underwent one or multiple IPPs.

### 3.8. Patient Health and Overall Satisfaction

Patient satisfaction with their IPP was generally high. A combined 87.93% of participants reported moderate to high satisfaction with their treatment: 44.83% (n = 78) expressed moderate satisfaction, while 43.10% (n = 75) reported high satisfaction. Only 12.07% (n = 21) indicated low satisfaction levels.

Perceived health status, measured by the EQ-5D Health scale, yielded similarly favorable outcomes. More than half of the participants (52.30%, n = 91) rated their health in the highest category (70–100), while 41.38% (n = 72) reported moderate health levels (40–60). Only 6.32% (n = 11) rated their perceived health condition as low (0–30), suggesting that most patients experienced positive health outcomes.

### 3.9. Patient Health Satisfaction in Relation to Sex

No significant differences were observed in the Patient Global Impression of Change (PGIC) scores between male and female participants following the first IPP. Both sexes had similar satisfaction levels (male median = 5, IQR = 3; female median = 5, IQR = 4; *p* = 0.8239). However, a statistically significant difference was noted in their perceived health status (EQ-5D Health), with female participants reporting higher median scores than males (female median = 75, IQR = 25 vs. male median = 70, IQR = 30; *p* = 0.03906). This finding may warrant further investigation to better understand potential sex-related variations in health perception

### 3.10. Patient Health Satisfaction in Relation to PT Compliance

Patient satisfaction, as measured by the PGIC, appeared higher in the PT-compliant group. Specifically, 56% of PT-compliant patients reported satisfaction compared to 44% in the non-compliant group. The median PGIC score for PT-compliant patients was 6 (IQR = 3), compared to 5 (IQR = 4) in the non-compliant group. However, this difference did not reach statistical significance (*p* = 0.1799).

### 3.11. Patient Health in Relation to Number of Procedures

Among patients who underwent only one IPP, 55% (62/113) reported satisfaction, while 45% (51/113) did not. In the group that received more than one IPP, 54% (33/61) were satisfied, and 46% (28/61) were not. When the PGIC scores were analyzed based on the number of procedures, the median satisfaction scores were comparable across groups, with no statistically significant differences (*p* = 1). Additionally, no significant sex differences were observed in the PGIC satisfaction scores or EQ-5D Health scores in relation to the number of procedures

## 4. Discussion

Interventional pain procedures (IPPs) and physical therapy (PT) have become essential components in the management of chronic back pain (CBP), offering both pain relief and functional improvements. However, our study highlights the complex interplay among the type of IPP, adherence to PT, analgesic use, and patient satisfaction—relationships that merit further analysis.

While the exact global volume of IPPs performed annually for back pain is unknown, it likely numbers in the millions and continues to grow. This trend is driven by the aging populations of many countries, an increased awareness of procedural options among patients, and advances in medical technology that have made IPPs safer and more accessible. When conducted in accordance with evidence-based guidelines, IPPs have a low risk profile: one large study involving 4209 lumbar IPPs reported no major adverse events and only a 1.4% rate of minor complications between 2015 and 2020 [16].

There is growing evidence supporting the efficacy of IPPs [17], yet few studies have provided long-term follow-up beyond the first year. Comprehensive clinical guidelines for IPPs have been published [18], but there remains a need for real-world data assessing long-term outcomes. For instance, one study involving 78 patients who received a course of single-level lumbar transforaminal epidural steroid injections for radicular pain due to disc herniation reported favorable outcomes at six months. However, symptoms often recurred within five years, necessitating additional treatments, such as repeat injections or alternative interventions [19].

Interventional pain procedures (IPPs) often provide immediate or near-immediate pain relief, with over 80% of patients reportedly benefiting from symptom reductions [20]. However, the long-term durability of this analgesic effect remains underexplored and appears to vary substantially across patient populations. For example, a longitudinal study involving 85 patients who underwent lumbar spinal surgery found no significant difference in visual analog scale (VAS) pain scores at 12 years between those treated with decompression alone versus those who underwent decompression with fusion and/or stabilization [21].

As IPPs for spinal disorders gain widespread adoption, especially due to their minimally invasive nature and relatively low risk, robust long-term outcome data are increasingly needed to guide clinical decision-making. Most IPP guidelines currently in use, including those cited in this study [6,8,9,17,22], originate from Western practice frameworks. Although our study was conducted in Qatar, the procedural standards followed were consistent with internationally accepted best practices for interventional pain care [23].

In the Middle East, pain medicine continues to emerge as a formal specialty. Additionally, societal perceptions regarding opioid use remain stigmatized, which may contribute to lower rates of analgesic prescribing compared to Western settings [23]. In this context, IPPs offer a compelling therapeutic alternative if they can effectively control pain and reduce long-term medication dependence. Despite this potential, our study did not demonstrate a statistically significant reduction in analgesic use following IPPs. Interestingly, a marginally significant difference was observed in medication consumption favoring PT-non-compliant patients. This counterintuitive trend aligns with findings by Sun et al., who reported that in patients with musculoskeletal pain, early engagement in physical therapy was associated with reduced long-term opioid use, suggesting that nonadherence may lead to greater medication reliance due to unresolved symptoms [24].

It is plausible that the patients who adhered to physiotherapy experienced temporary discomfort or increased pain due to physical exertion, which may have led to higher short-term analgesic use. In contrast, the non-compliant patients may have avoided such transient pain by limiting physical activity, thereby altering their medication patterns. Beyond PT adherence, psychological factors—particularly pain catastrophizing—have been shown to influence IPP outcomes. Rajput et al. [25] found that higher preprocedural Pain Catastrophizing Scale (PCS) scores were associated with greater emotional interference from pain. Reductions in pain severity post-procedure were linked to reduced catastrophizing, suggesting that this psychological trait is modifiable. Notably, emotional distress, rather than baseline pain intensity, was the strongest predictor of follow-up PCS scores.

Our findings similarly revealed no significant difference in analgesic use between PT-compliant and non-compliant patients, despite early pain relief being more pronounced among the compliant group. This raises the possibility that psychosocial variables, such as catastrophizing or perceived burden of therapy, may contribute to long-term medication use and treatment outcomes. Future studies should explore whether integrating psychological or behavioral interventions into post-IPP rehabilitation might optimize outcomes—particularly in patients with high baseline emotional distress or maladaptive pain responses [25].

PT adherence is often suboptimal in real-world practice. In our cohort, patients who completed their prescribed PT showed significantly improved pain scores at six months post-IPP, but these benefits diminished over time. As PT in this study was delivered in a finite course, most patients discontinued therapy once the program ended. These findings suggest that repeated or long-term PT engagement may be necessary to sustain improvement. Consistent with prior research, PT adherence has been linked to factors such as patient motivation, self-efficacy, and beliefs about treatment value [26]. Future research should evaluate the ideal frequency and duration of PT to maintain benefits following IPP, as well as how psychosocial support can enhance long-term adherence.

It remains unclear from our study why some patients did not participate in PT. A cross-sectional study of orthopedic surgery patients in Saudi Arabia found that a younger age, male sex, and unmarried status were risk factors for noncompliance with PT regimens. This study also found that patients were significantly more likely to skip PT sessions once they noticed symptomatic improvement [27]. This does not imply that effective PT itself promotes noncompliance, but rather that patient education may be needed to help post-procedure patients understand that improvements achieved through PT are not endpoints, but a status that must be actively maintained through continued sessions [14].

It has been shown that self-efficacy and patient motivation are strong predictors of adherence to PT, including home-based programs, although these variables were not measured in our study [17,27]. Furthermore, PT adherence is likely influenced by the patient’s baseline physical activity level, acceptance of and perceived benefit from exercise, social support, and the presence of anxiety or depression [28,29].

The degree to which pain during physiotherapy (PT) may impact adherence has not been well studied. In our study, all participants had access to expert PT services; however, this is not the case for all patients undergoing an IPP for back pain. In many countries with public healthcare systems, PT access is often limited due to high patient volumes, long waiting lists, and constrained resources [30]. As a result, low PT compliance may not always reflect patient preferences, but instead structural barriers, such as delayed treatment initiation and financial limitations—both of which can significantly affect rehabilitation outcomes [31].

The long-term nature of our study provides valuable insights, though it may also introduce confounding factors. Patients were followed for four years after their initial IPP, during which time they may have experienced new health issues, comorbidities, or general health deterioration. The mean age in our cohort was 56.71 ± 13.07 years for men and 51.88 ± 12.26 years for women. While many patients may have experienced early positive outcomes from their IPP and their prescribed PT course, later health problems could have reintroduced pain. Thus, the diminishing long-term impact of PT may be more related to the progression of spinal degenerative disease than to the efficacy of PT itself. Moreover, the prevalence of chronic pain increases with advancing age, meaning long-term studies like ours are inherently influenced by the higher likelihood of pain recurrence or emergence over time [32,33].

While IPPs are generally effective in relieving pain, our study found that patients who underwent only one IPP reported slightly higher satisfaction (55%) compared to those who received multiple procedures (54%). Although this difference was not statistically significant, it may suggest that repeated interventions do not necessarily yield proportional improvements. Potential explanations include diminishing returns, varying levels of patient resilience, and differing expectations.

This study has several limitations. First, we enrolled patients with various spinal pathologies and treated them with three different types of IPPs. In some analyses, we grouped these procedures together, which may have obscured nuanced effects specific to each intervention. Second, all participants were drawn from a single healthcare system in Qatar, which may limit the generalizability of the findings to other populations or healthcare environments.

Third, only patients who completed the full 48-month follow-up were included in the analysis. Dropout reasons were not investigated. For instance, patients who did not benefit from an IPP may have withdrawn, potentially skewing the outcome data. Fourth, although obesity has been shown to influence the risk profile for certain IPPs, we did not stratify patients based on body mass index (BMI), which may be an important variable for future research [34]. Finally, this study did not continuously monitor all possible health developments over the four-year period, such as new comorbidities, injuries, or age-related musculoskeletal changes, which may have affected long-term outcomes.

Despite these limitations, one of the key strengths of our study is the structured long-term follow-up. Patients were evaluated every six months during the first year and then annually for four years, providing a comprehensive dataset on the durability of treatment effects over time.

## 5. Conclusions

This study followed 174 patients with chronic back pain who underwent one of three interventional pain procedures (IPPs) at a single center in Qatar, with structured follow-up over four years. Pain scores, disability, analgesic use, patient satisfaction, and physical therapy (PT) compliance were evaluated. Although no significant difference in analgesic use was observed between PT-compliant and non-compliant groups, most patients reported satisfaction with their outcomes. Approximately 57% reduced medication use, while 43% increased it. Notably, 55% of patients required only one IPP during the study period, and no serious adverse events were reported. These findings support the effectiveness and safety of IPPs in managing chronic back pain. However, further large-scale, multicenter research is warranted to explore long-term outcomes and optimize post-procedural rehabilitation strategies.

## Figures and Tables

**Table 1:** Demographic and clinical characteristics of study participants.

Characteristics	n	%
**Sex**		
Male	99	56.9
Female	75	43.1
**Age**		
Male	56.71 ± 13.07	
Female	51.88 ± 12.26	
**Ethnicity**		
Asian	33	19.0
Middle Eastern	102	58.6
African	32	18.4
European	7	4.0
**Type of Procedures**		
Facets (MBB diagnostic/ablation)	67	38.5
Transforaminal epidural steroid block	61	34.9
Sacroiliac joint (diagnostic/ablation)	46	26.4
**No. of Procedures Undergone**		
One only	113	64.9
>1 (Same procedure repeated)	61	35.1
**Medication**		
Increased	75	43.1
Decreased	99	56.9
**Physical Therapy**		
Compliant	68.0	39.1
Not compliant	106.0	60.9

**Table 2:** Summary of median, IQR, and *p* values for NRS, EQ5D, and ODI scores over time.

Metric	Time	Median	IQR	*p* Value
EQ5D	pre	0.4945	0.23875	BASELINE
EQ5D	6 mo	0.752	0.179	<0.001
EQ5D	12 mo	0.7505	0.179	<0.001
EQ5D	24 mo	0.7415	0.18325	<0.001
EQ5D	36 mo	0.74	0.1935	<0.001
EQ5D	48 mo	0.744	0.18825	<0.001
NRS	pre	8	2	BASELINE
NRS	6 mo	5	5	<0.001
NRS	12 mo	5	5	<0.001
NRS	24 mo	4	4	<0.001
NRS	36 mo	4	4	<0.001
NRS	48 mo	4.5	4	<0.001
ODI	pre	44	24	BASELINE
ODI	6 mo	25.5	18.75	<0.001
ODI	12 mo	26.5	19.75	<0.001
ODI	24 mo	29	24	<0.001
ODI	36 mo	29.5	30.5	<0.001
ODI	48 mo	28.5	35	<0.001

EQ5D 5D, Euro Qual Index; mo, month(s); NRS, numeric rating scale; and ODI, Oswestry Disability Index.

**Table 3:** A comparison between PT-compliant and non-compliant patients at various time points of this study.

Measure	Time Comparison	Group	W	*p* Value
NRS	Baseline vs. 6 mo	Compliant	1756.5	<0.0001
		Non-compliant	1756.5	<0.0001
	6 vs. 12 mo	Compliant	505.5	0.257
		Non-compliant	505.5	0.257
	12 vs. 24 mo	Compliant	938.5	0.0232
		Non-compliant	938.5	0.0232
	24 vs. 36 mo	Compliant	395.5	0.644
		Non-compliant	395.5	0.644
	36 vs. 48 mo	Compliant	676.5	1
		Non-compliant	676.5	1
EQ5D	0 vs. 6 mo	Compliant	1267	<0.0001
		Non-compliant	1267	<0.0001
	6 vs. 12 mo	Compliant	694.5	0.834
		Non-compliant	694.5	0.834
	12 vs. 24 mo	Compliant	487.5	0.429
		Non-compliant	487.5	0.429
	24 vs. 36 mo	Compliant	235.5	0.0133
		Non-compliant	235.5	0.0133
	36 vs. 48 mo	Compliant	1056.5	0.0427
		Non-compliant	1056.5	0.0427
ODI	0 vs. 6 mo	Compliant	1364.5	<0.0001
		Non-compliant	1364.5	<0.0001
	6 vs. 12 mo	Compliant	283.5	0.0402
		Non-compliant	283.5	0.0402
	12 vs. 24 mo	Compliant	1356.5	0.0594
		Non-compliant	1356.5	0.0594
	24 vs. 36 mo	Compliant	224.5	0.00497
		Non-compliant	224.5	0.00497
	36 vs. 48 mo	Compliant	678.5	0.362
		Non-compliant	678.5	0.362

EQ, Euro Qual Index; NRS, numeric rating scale; and ODI, Oswestry Disability Index. W = Wilcoxon Signed-Rank Test statistic.

**Table 4:** A comparison between a single IPP and >1 IPP.

Measure	Time	>1 IPP Median	One IPP Median	*p* Value
NRS	0	7.5	8	0.114
NRS	6	4.5	4.5	0.795
NRS	12	4.5	4.5	0.984
NRS	24	4.6	4.5	0.858
NRS	36	4.5	4.5	0.889
NRS	48	4.88	5	0.99
EQ5D	0	0.518	0.463	0.149
EQ5D	6	0.722	0.738	0.376
EQ5D	12	0.723	0.738	0.31
EQ5D	24	0.727	0.739	0.386
EQ5D	36	0.711	0.73	0.44
EQ5D	48	0.738	0.741	0.47
ODI	0	43.2	44	0.625
ODI	6	28.8	26	0.386
ODI	12	29.8	26	0.406
ODI	24	30.2	29.5	0.454
ODI	36	31.5	31.5	0.532
ODI	48	30.8	30	0.555

**Table 5:** Dunn’s test results.

Group 1	Group 2	Z Value	P_unadj	P_adj
PT-compliant	One IPP	3.610	0.058	0.173
PT-compliant	>1 IPP	4.610	0.032	0.095
PT non-compliant	One IPP	0.020	0.884	1.000
PT non-compliant	>1 IPP	0.280	0.597	1.000

## Data Availability

The data supporting the findings of this study are not publicly available due to institutional privacy policies and ethical restrictions at Hamad Medical Corporation (HMC), which prohibit the sharing of patient-related clinical data. Access to data may be considered upon reasonable request and with appropriate institutional approvals.

## Abbreviations

The following abbreviations are used in this manuscript:

IPP	Interventional Pain Procedures
NRS	Numeric Rating Scale
ODI	Oswestry Disability Index
EQ5D	EuroQol 5Dimension Index
EQHealth	EuroQol Health Status Visual Analog Scale
PGIC	Patient Global Impression of Change
PT	Physical Therapy
IQR	Interquartile Range
